# Using Next Generation Chemiluminescent Probes to Improve the *Plasmodium falciparum* *in vitro* Parasite Reduction Ratio (PRR) Assay

**DOI:** 10.1021/acsinfecdis.5c00924

**Published:** 2026-01-23

**Authors:** Angela Hellingman, Nele Lara Göttle, Annabelle Walz, Nicolas Michel Beat Brancucci, Sergio Wittlin, Pascal Mäser, Matthias Rottmann

**Affiliations:** † 30247 Swiss Tropical and Public Health Institute, 4123 Allschwil, Switzerland; ‡ University of Basel, 4001 Basel, Switzerland

**Keywords:** malaria, P. falciparum, PRR assay, optimization, chemiluminescence, AquaSpark

## Abstract

Malaria remains a major global health threat, and the emergence of partial artemisinin resistance challenges current treatment regimens. Reliable antimalarial screening assays are therefore essential for identifying new drug candidates. The parasite reduction ratio (PRR) assay provides valuable pharmacodynamic insights but is limited by its labor-intensive, 14- to 28-day incubation period. We developed an optimized PRR assay protocol using the highly sensitive chemiluminescence-based *lacZ*/β-gal^SENSOR^ readout, reducing assay incubation duration to 7 days while maintaining informative pharmacodynamic parameters, including lag phase, parasite clearance time, parasite reduction ratio, and maximum killing effect. In contrast, the [^3^H]-hypoxanthine incorporation method failed to detect viable parasites reliably and consistently overestimated drug activity with the shortened protocol. This novel *lacZ*/β-gal^SENSOR^ PRR assay enables laboratories without access to radioactive facilities to evaluate antimalarial compounds efficiently, providing robust time–killing profiles with greater convenience, higher throughput, and lower equipment requirements than existing readout methods.

## Graphical abstract

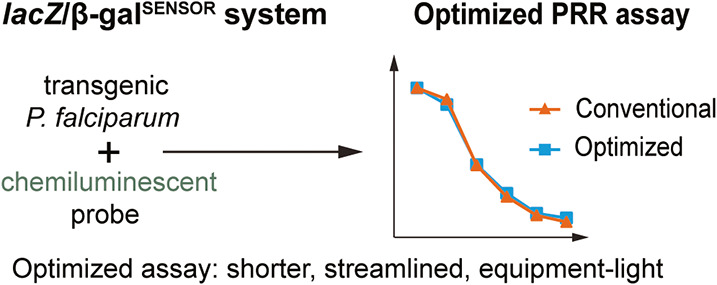

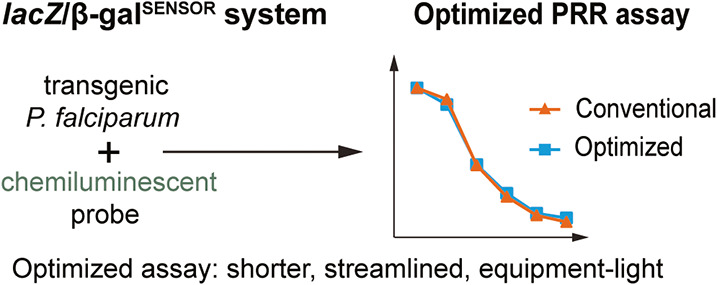

Malaria is a vector-borne parasitic disease transmitted by female *Anopheles* mosquitoes. Nearly half of the world population lives in malaria-endemic regions, and in 2024, an estimated 282 million cases and 610000 deaths occurred worldwide.^1,2^ *Plasmodium falciparum* is the most virulent malaria parasite, resulting in high mortality if left untreated.^2,3^ First-line treatments against a *P. falciparum* infection are the artemisinin-based combination therapies. However, partial artemisinin resistance is increasing and threatening antimalarial chemotherapy.^4–9^ Therefore, the development of new antimalarial drugs and combination regimens is of great importance.^6^

*In vitro* drug susceptibility assays are crucial tools to discover and characterize novel antiplasmodial molecules and promote their further development. The half-maximal inhibitory concentration (IC_50_) is a key parameter that indicates the potency of drug candidates. The disadvantage of conventional IC_50_ assays in antimalarial drug discovery is that all of them, regardless of the readout method, measure parasite viability in an indirect way.^10–12^ Discriminating between cytotoxic or cytostatic compounds is hardly possible. Overestimation of antiplasmodial potency can be caused by viable but metabolically inactive parasites; underestimation by parasites that are committed to death but still show metabolic activity; compounds that directly affect the used readout method can cause artifacts. Furthermore, IC_50_ assays do not measure the onset- and speed-of-action of the tested compounds, which in the case of malaria are key to successful treatment giving the fast proliferation of the parasites in a patient.^10,11,13^

To directly assess parasite viability, Sanz et al. developed the *in vitro* parasite reduction ratio (PRR) assay.^11^ This method quantifies the number of viable parasites remaining after drug exposure by performing limiting dilutions and allowing any surviving parasites to regrow. Parasite viability at the end of the drug exposure period is then inferred from the initial parasite number per well, the dilution factor, and the number of titer levels that show parasite regrowth. In brief, the experimental procedure is as follows: Parasites are treated with the desired compound for up to 120 hours (h) (Figure 1 first point). For each time point tested, an aliquot of treated parasites is collected, the drug is removed by washout, and the sample is serially diluted in fresh medium and red blood cells (Figure 1 second and third point). The cultures are incubated for 21–28 days (Figure 1 fourth point). Finally, growth of the parasites can be assessed by the incorporation of [^3^H]-hypoxanthine^11^ (Figure 1 fifth point). This protocol enables to extrapolate the number of viable parasites after drug treatment, and to establish a time-killing profile which informs about important *in vitro* pharmacodynamic (PD) parameters such as (1) the parasite reduction ratio, which is the parasitemia ratio between 0 and 48 h of drug treatment; (2) the parasite clearance time after which 99.9% of the initial parasites have been cleared (PCT_99.9%_); (3) the lag time, which describes the time until the maximum killing rate is achieved by the tested compound; and (4) the maximum effect (*E*_max_), which describes the maximum killing rate of the tested drug^11^ (Figure 1 sixth point).

**Figure 1:**
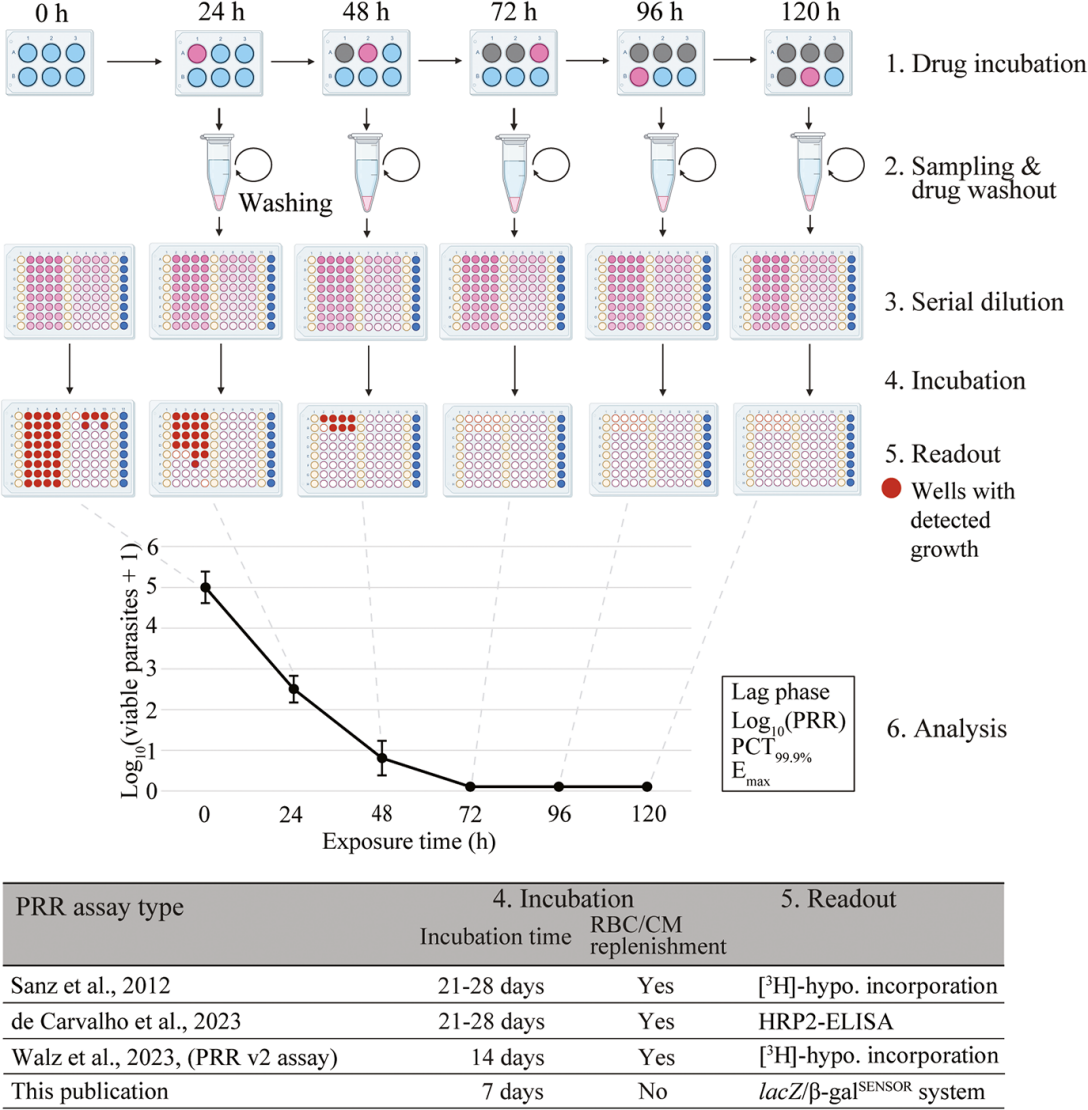
Schematic representation of the PRR assay protocol. (1) The parasites are treated with the test compound for different time periods. (2) The compound is then removed by several rounds of washout. (3) The washed sample is serially diluted, and (4) any viable parasites remaining after treatment are given a recovery time of up to 28 days. (5) Viable parasites are measured using [^3^H]-hypoxanthine incorporation, HRP-2 ELISA or the chemiluminescent *lacZ/*β-gal^SENSOR^ reporter system. (6) Pharmacodynamic parameters are derived in a semiautomated analysis. PRR = parasite reduction ratio, PCT = 99.9% parasite clearance time (h), *E*_max_ = maximum effect (h^–1^), CM = culture medium, RBC = red blood cells, hypo. = hypoxanthine, HRP-2 ELISA = histidine-rich protein 2 sandwich enzyme-linked immunosorbent assay. Figure 1 was partly created in BioRender. Rottmann, M. (2026) https://BioRender.com/5l3iur7.

This assay has become a mainstay in the preclinical evaluation of candidate antimalarials. The main disadvantage of this methodology is that it is work-intensive, time-consuming, and expensive. In addition, the [^3^H]-hypoxanthine incorporation readout requires specialized laboratory equipment and environment for handling radioactive material. Walz et al. developed the PRR version 2 (PRR v2) assay, reducing the parasite recovery incubation period from 21–28 days to 14 days.^14^ However, this still required the replenishment of red blood cells (RBCs) and culture medium (CM) during the incubation period, increasing the risk of microbiological or well-to-well contamination. De Carvalho et al. adapted the PRR assay for the histidine-rich protein 2 sandwich enzyme-linked immunosorbent assay (HRP-2 ELISA) readout, making it also independent of radioactive readout.^15^ They tested a shorter recovery incubation period and found that assessing viability after only 7 days could lead to an overestimation of slow-acting drugs’ effects.^15^ In summary, shortening the recovery period in PRR assay protocols is constrained by the sensitivity of the readout method.

To address this experimental challenge, the chemiluminescence-based *lacZ*/β-gal^SENSOR^ reporter system offers a viable solution, providing a highly sensitive readout method, enabling immediate detection of around 200 parasites per well under assay conditions. This system can be applied both in a conventional IC_50_ assay, as well as a PRR assay with a 14-day parasite recovery period.^16^ This method opens new possibilities for reducing assay duration and workload. Here we evaluate whether it can be applied to optimize the PRR assay by shortening the parasite recovery incubation time from 14 to 7 days. This would decrease the workload, minimize the risk of contamination, eliminate the need for radioactivity, and speed up the drug discovery process.^16^

## Results and Discussion

Using the highly sensitive *lacZ*/β-gal^SENSOR^ system, we aimed to create a PRR assay protocol with a 7-day recovery time, based on the PRR v2 assay protocol,^14^ adapted for our chemiluminescent readout method. In this method, parasite viability is determined by the production and activity of the reporter enzyme β-galactosidase, which activates the sensor probe resulting in a chemiluminescent signal.^16^ As the β-galactosidase reporter enzyme produced by the transgenic F12^*lacZ*^ parasites remains stable for up to 28 days under cultivation conditions,^16^ two negative controls were implemented to ensure reliable distinction between signal derived from viable parasite regrowth and residual reporter enzyme activity present before or during drug treatment: (i) uninfected RBCs to correct for signal-to-background noise, and (ii) a frozen, undiluted, drug-treated parasite sample collected immediately after drug washout to account for the amount of stable β-galactosidase present at the end of drug exposure.

To assess assay performance, we compared results obtained with the 7-day chemiluminescent PRR assay protocol to results originating from the 14-day chemiluminescent PRR assay protocol, the 7-day [^3^H]-hypoxanthine incorporation PRR assay readout and to published values of the PRR v2 assay (14-day recovery time).^14^ Four reference antimalarials with known speeds of action – artemisinin, chloroquine, pyrimethamine, and atovaquone – were tested in parallel in at least three biological replicates using the transgenic F12^*lacZ*^ strain. Final results were normalized to correspond to 10^5^ parasites at 0 h. PD parameters (log_10_(PRR), lag time, and PCT_99.9%_) were derived via the R-based analysis pipeline of Walz et al.^14^

The 7-day [^3^H]-hypoxanthine incorporation PRR assay correctly classified atovaquone as slow-acting and artemisinin and chloroquine as fast-acting, while pyrimethamine exhibited a fast-acting profile with lag phase. However, the shortened [^3^H]-hypoxanthine protocol tended to overestimate drug activity—particularly for fast-acting compounds—and often failed to detect viable parasites at time points where the PRR v2 assay still indicated regrowth (Figure 2). This reduced sensitivity of the 7-day [^3^H]-hypoxanthine incorporation readout is clearly evident in the not normalized time-killing profiles, requiring an average normalization factor of 2.8 for normalization to 10^5^ parasites at 0 h. This indicates that after 7 days of incubation, the assay underestimated the actual starting inoculum of 10^5^ parasites.

**Figure 2:**
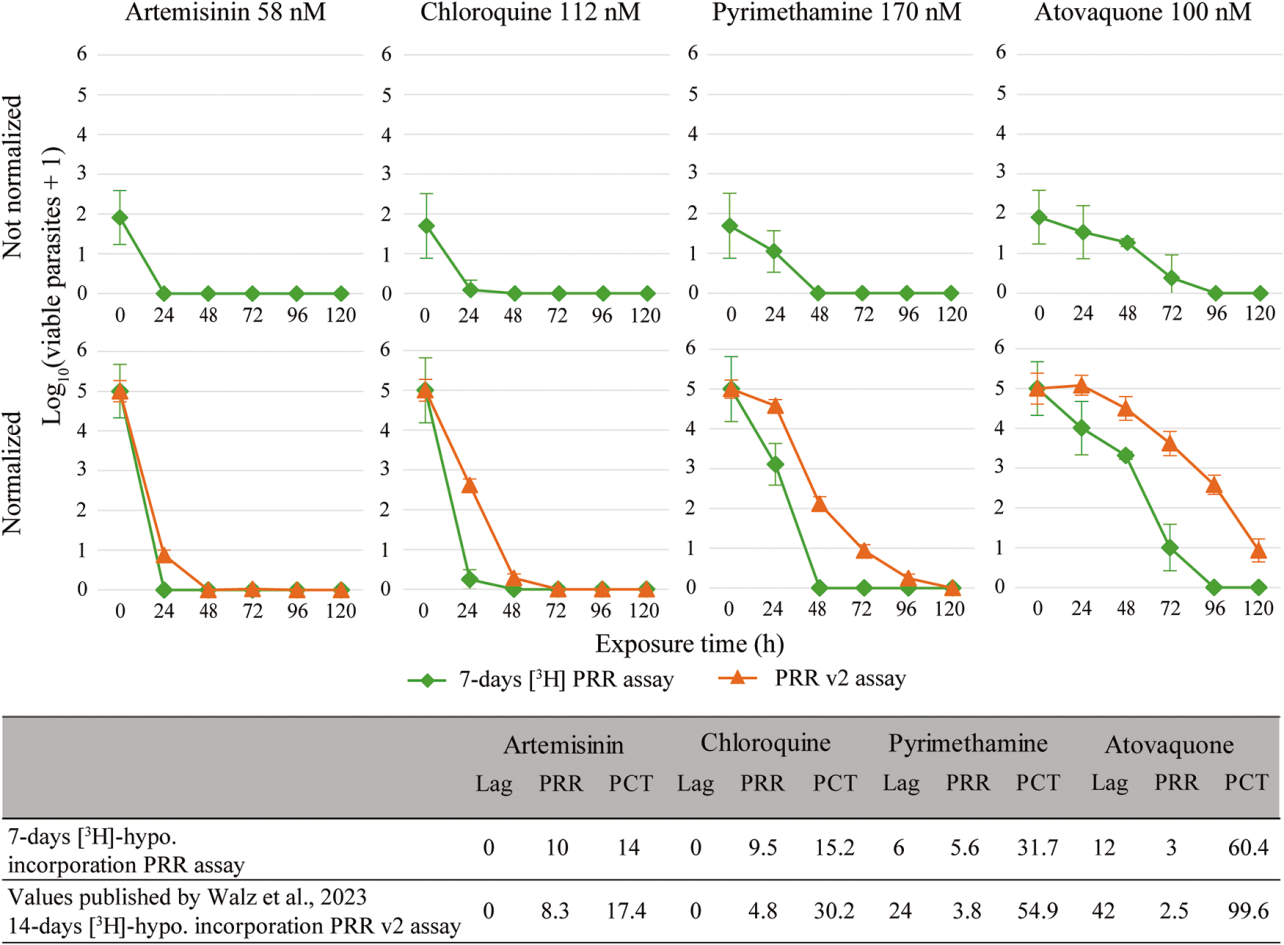
Not normalized and normalized time-killing profiles of the four reference drugs of the published PRR v2 assay vs the 7-day [^3^H]-hypoxanthine readout PRR assay, and published or calculated pharmacodynamic parameters respectively: Lag = lag time (h), PRR = parasite reduction ratio and PCT = 99.9% parasite clearance time (h). The four reference drugs - artemisinin (58 nM), chloroquine (112 nM), pyrimethamine (170 nM) and atovaquone (100 nM) - were tested in at least three independent experiments. Error bars represent the standard error of the mean (SEM) of these experiments performed in technical quadruplicates.

In contrast, not normalized time-killing profiles of the 7-day *lacZ*/β-gal^SENSOR^ probe PRR assay indicate superior sensitivity compared to the 7-day [^3^H]-hypoxanthine incorporation readout and only slightly lower sensitivity than the 14-day assay, with average normalization factors of 1.2 and 1.0, respectively. Across parallel experiments, the normalized 14-day and 7-day *lacZ*/β-gal^SENSOR^ probe PRR assay, as well as the published PRR v2 assay, produced overlapping time-killing profiles (Figure 3). The 7-day *lacZ*/β-gal^SENSOR^ probe PRR assay yielded slightly steeper time-killing profiles than the 14-day version, consistent with calculated PD parameters (Table 1). Atovaquone was classified as slow-acting in both assays, artemisinin and chloroquine as fast-acting, and pyrimethamine as intermediate-acting with lag phase in the 7-day assay but slow-acting in the 14-day assay. Overall, PD parameters derived from the 7-day *lacZ*/β-gal^SENSOR^ probe PRR assay showed closer agreement with published reference data than those from the 14-day chemiluminescent assay. Minor deviations were observed for slow-acting compounds such as atovaquone, for which the 7-day assay underestimated the lag time and produced the lowest log_10_(PRR) value among the three readout methods. For pyrimethamine, the log_10_(PRR) value resulted in a different classification of speed of action, as mentioned previously. Most importantly, the 7-day assay detected viable parasites at all time points as the 14-day assay, and produced overlapping time-killing profiles. The average growth rate of *P. falciparum* F12^*lacZ*^, determined by microscopy, was 0.048 per hour (natural logarithm scale; standard deviation (SD) 0.001), which corresponds to 0.021 per hour (log_10_ scale; SD 0.0006).

**Figure 3:**
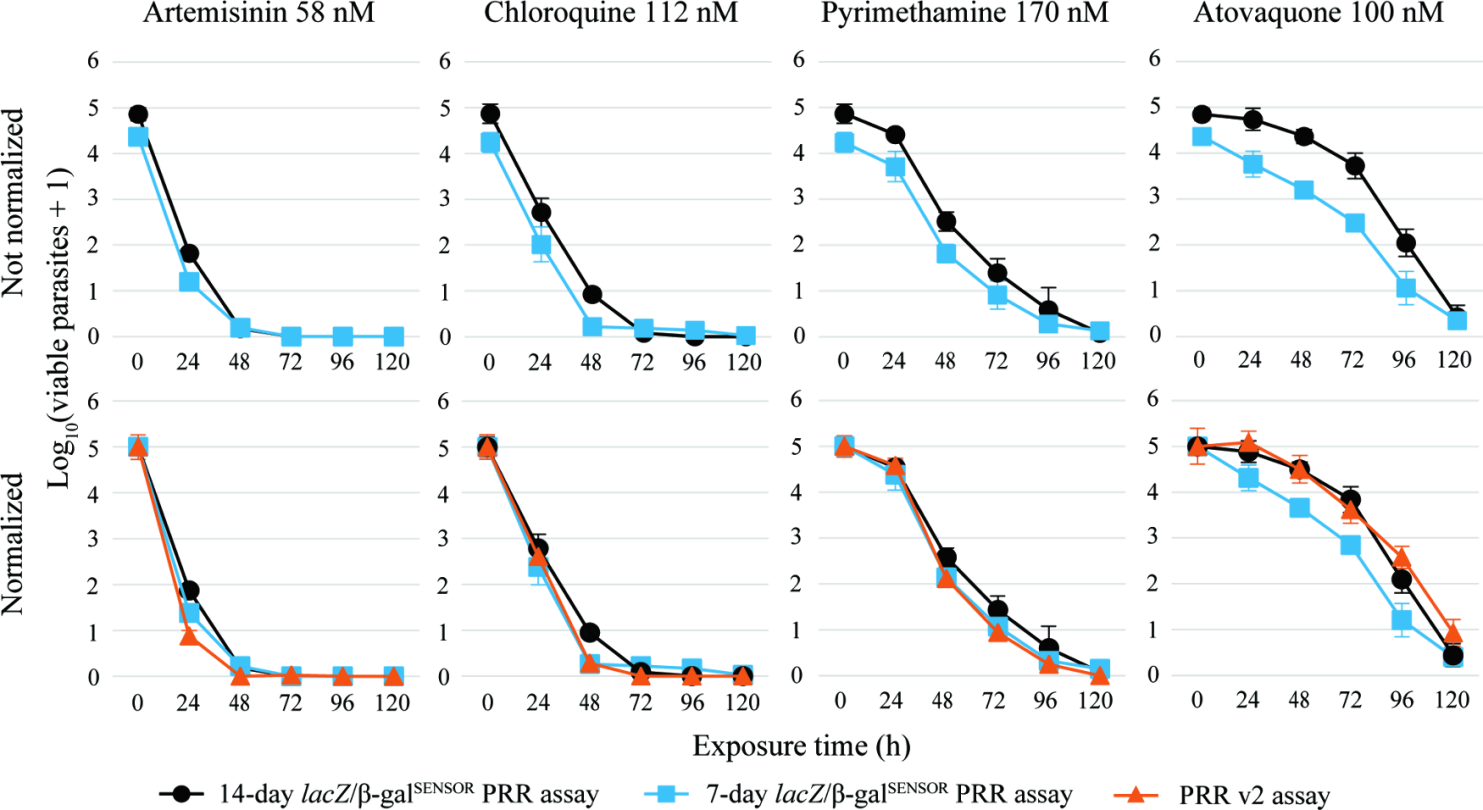
Comparison of the 14-day vs 7-day *lacZ*/β-gal^SENSOR^ probe PRR assay in comparison to the published PRR v2 assay. The four reference drugs artemisinin (58 nM), atovaquone (100 nM), chloroquine (112 nM) and pyrimethamine (170 nM) were tested in at least three independent experiments. Time-killing profiles were normalized with error bars representing the standard error of the mean (SEM) of the independent experiments in technical quadruplicates.

**Table 1:** Summary Table of Calculated Pharmacodynamic Parameters^a^

		*lacZ*/β-gal^SENSOR^ probe PRR assay				[^3^H]-hypo. incorporation PRR v2 assay (Walz et al.)	
Drugs	Parameters	7-days		14-days		14-days	
							
Artemisinin	Lag time [h]	0		0		0	
	Log_10_(PRR)	7.3	[6.9–7.7]	6.3	[6–6.6]	8.3	[8.0–8.6]
	PCT_99.9%_ [h]	19.9	[18.9–20.9]	23	[22–24.2]	17.4	[16.8–18]
	E_max_ [h^–1^]	0.4	[0.38–0.42]	0.35	[0.34–0.36]	0.44	[0.43–0.46]
	Speed of action	fast		fast		fast	
							
Chloroquine	Lag time [h]	0		0		0	
	Log_10_(PRR)	4.8	[4.5–5.2]	4.1	[3.9–4.4]	4.8	[4.6–4.9]
	PCT_99.9%_ [h]	29.8	[27.9–31.9]	34.9	[32.9–37.2]	30.2	[29.4–31.1]
	E_max_ [h^–1^]	0.28	[0.27–0.3]	0.25	[0.23–0.26]	0.27	[0.27–0.28]
	Speed of action	fast		fast		fast	
							
Pyrimethamine	Lag time [h]	12	[0–24]	12	[0–24]	16.8	[0–24]
	Log_10_(PRR)	3.3	[3–3.6]	2.7	[2.5–2.9]	3.8	[3.4–4.2]
	PCT_99.9%_ [h]	55.7	[51.9–60.2]	65.6	[61.4–70.6]	54.9	[51.3–59.4]
	E_max_ [h^–1^]	0.21	[0.19–0.22]	0.18	[0.17–0.19]	0.23	[0.21–0.25]
	Speed of action	intermediate with lag phase		slow		Intermediate with lag phase	
							
Atovaquone	Lag time [h]	12	[0–24]	54	[48–72]	42	[24–48]
	Log_10_(PRR)	2	[1.9–2.2]	3.3	[3–3.6]	2.5	[1.7–3.4]
	PCT_99.9%_ [h]	83.2	[78.7–88.4]	97.6	[94.3–101.5]	99.6	[84.9–129.5]
	E_max_ [h^–1^]	0.15	[0.14–0.15]	0.21	[0.19–0.22]	0.17	[0.13–0.21]
	Speed of action	slow		slow		slow	

a The 95% confidence interval of pharmacodynamic parameters generated in at least three biological replicates is shown in square brackets. PRR = parasite reduction ratio, PCT = 99.9% parasite clearance time (h), *E*_max_ = maximum effect (h^–1^).

Unlike the 14-day PRR v2 assay, which requires two RBC replenishment steps, the 7-day *lacZ*/β-gal^SENSOR^ probe PRR assay protocol omits these steps while maintaining discriminatory power. Reducing recovery time thereby simplifies the workflow by eliminating washing and RBC replenishment after the first week of drug incubation and sampling, which not only mitigates contamination risk but also substantially reduces experimental complexity. Importantly, the chemiluminescent *lacZ*/β-gal^SENSOR^ readout further increases accessibility, as it provides rapid and straightforward quantification without processing requirements needed for [^3^H]-hypoxanthine incorporation or HRP-2 ELISA methods.^14,15^ For signal quantification, a standard plate reader capable of measuring luminescence is sufficient.

The *lacZ*/β-gal^SENSOR^ system relies on the accumulation of a stable reporter enzyme that is constitutively produced by metabolically active transgenic parasites. As the enzyme and its activity remain stable, the system still generates a signal throughout the recovery period. This enzymatic persistence ensures continuous signal generation throughout the recovery phase, enabling detection of parasite overgrowth. However, it should be noted that 7-day versus 14-day enzyme accumulation can produce different time-killing profiles for certain drugs that potentially affect parasite metabolism after treatment. This may partly explain the differences in the observed lag phase of atovaquone between the 7-day and 14-day PRR assays. For compounds that result in very low numbers of surviving parasites and alter parasite metabolism the 7-day *lacZ*/β-gal^SENSOR^ probe PRR assay may miss some information, as full recovery and reaching the limit of detection may not be achieved within the 7-day incubation period. In contrast, the [^3^H]-hypoxanthine incorporation method only assesses viability for the final 24 h after [^3^H]-hypoxanthine is added, so it cannot detect overgrowth during this period and relies on a secondary readout, e.g., the discoloration of the filter mats during harvesting of the parasites. While the *lacZ*/β-gal^SENSOR^ system offers significant advantages in sensitivity, simplicity, and nonradioactive detection, its main limitation remains its reliance on transgenic parasites.^16^ At present, no commercially available chemiluminescent probe is specific for *Plasmodium* detection, which restricts the use of this system to*P. falciparum* strains engineered to express the *lacZ* gene. For rapid testing of field isolates, direct viability assessment using MitoTracker Deep Red could be an alternative, though it requires costly flow cytometry and precludes frozen storage prior to analysis.^17^

## Conclusion

In conclusion, the novel *lacZ*/β-gal^SENSOR^ PRR assay protocol presented here provides a highly sensitive and practical tool for classification of antimalarials during drug discovery and development. By shortening the incubation period to 7 days and eliminating reliance on radioactive materials or complex instrumentation, this method offers a sensitive, scalable, and user-friendly platform suitable to quantify drug activity for any laboratory capable of cultivating *Plasmodium* parasites.

## Material and Methods

### *Plasmodium falciparum* *in vitro* Cultivation

The*P. falciparum*strain F12^*lacZ*^ ^16^ was maintained in continuous culture at a hematocrit of 5% and incubated at 37 °C in gas chambers containing a gas mixture composed of 93% N_2_, 4% CO_2_, and 3% O_2_ as proposed by standard methods.^18,19^ The culture medium (CM) used consisted of RPMI 1640 (10.44 g/L, ThermoFisher Scientific), supplemented with 25 mM HEPES (5.94 g/L, Sigma-Aldrich), NaHCO_3_ (2.1 g/L, Sigma-Aldrich), neomycin (100 mg/L, Sigma-Aldrich), Albumax II (5 g/L, Thermo Fisher), and 0.36 mM hypoxanthine (50 mg/L, Sigma-Aldrich). The ingredients were dissolved in water (Milli-Q purified), mixed for 3 h or more and sterile filtered with a bottle top filter (0.22 μm pore size, Corning). Human erythrocyte concentrates were obtained from the local blood bank.

### IC_50_ Assay: [^3^H]-Hypoxanthine Incorporation

Reference compound stock solutions were prepared by dissolving compound powder in dimethylsulfoxide (DMSO) (artemisinin, atovaquone, pyrimethamine) or purified, sterile filtered water (chloroquine) respectively, to obtain a drug concentration of 10 mM. To validate prepared compound stock solutions, [^3^H]-hypoxanthine incorporation IC_50_ assays were performed according to the protocol published by Snyder et al.^19^ Briefly, compounds were serially diluted in hypoxanthine deficient medium in 96-well plates. Infected red blood cells were added (final hematocrit of 1.25% and 0.3% parasitemia) and the assay plates were incubated for 48 h under cultivation conditions in humidified gas chambers. After 48 h, tritium labeled hypoxanthine was added and the assay plates were incubated for another 24 h. Radioactivity of incorporated tritium was measured using a Betaplate liquid scintillation counter. IC_50_-values were estimated using linear interpolation^19,20^ and the resulting values were compared with previously published data.^21^

### Parasite Reduction Ratio Assay

The published protocol for the PRR v2 assay by Walz et al.,^14^ which was adapted from Sanz et al.,^11^ formed the basis of the work presented here.

Asexual, unsynchronized *P. falciparum* parasites (transgenic strain F12^*lacZ*^)^16^ with a hematocrit of 1.25% and parasitemia of 0.3% (with at least 60% ring stages) were distributed into 6-well plates and incubated under cultivation conditions either with CM alone (growth control) or under drug pressure (10 × IC_50_ concentration for artemisinin, chloroquine and pyrimethamine; 100 nM for atovaquone). For the growth control, blood smears were prepared after 48 h of incubation, after which the parasitemia was determined in order to calculate the growth rate of the parasite culture. For drug-treated cultures, drug and CM was replaced every 24 h. From 0 up to 120 h, an aliquot of 5.5 mL was taken every 24 h and the drug was removed by three washing cycles: centrifugation (600 g, 2 min), removing supernatant and addition of 5.5 mL fresh CM. The three different PRR assays were initiated with the same washed aliquot (same starting conditions): assay 1 - the 7-day [^3^H]-hypoxanthine incorporation PRR assay (7-day parasite recovery time), assay 2 - the 7-day *lacZ*/β-gal^SENSOR^ probe PRR assay (also 7-day parasite recovery time) and assay 3 - the 14-day *lacZ*/β-gal^SENSOR^ probe PRR assay (14-day parasite recovery time).

Four technical replicates (eight technical replicates for time point 0 h, each corresponding to a theoretical parasite number of 10^5^ parasites per well if untreated) of the washed aliquots were distributed into 96-well plates (transparent plates for assay 1, Sarstedt; white plates for assay 2 and 3, Greiner Bio-One) and serially diluted 15 times with a dilution factor of 4. In addition, from each washed aliquot, two control aliquots were taken and frozen at −20 °C. The 96-well plates were incubated in a humidified gas chamber under cultivation conditions for 7 days (assay 1 and 2) or 14-days (assay 3). For the 14-day PRR assay, once a week supernatant was removed and replaced with fresh erythrocytes at a hematocrit of 1.5%. For the 7-day [^3^H]-hypoxanthine incorporation PRR assay (assay 1)), 6 days after serial dilution CM was replaced with 0.5 μCi of [^3^H]-hypoxanthine in hypoxanthine deficient medium, incubated for another 24 h and then frozen at – 20 ◦C. Seven or 14 days after the serial dilution of the 7-day (assay 2) or 14-days (assay 3) *lacZ*/β-gal^SENSOR^ probe PRR assay plates were frozen at −20 °C.

For signal measurement, the assay plates and the two frozen aliquots of the washed samples (one for assay 2 and one for assay 3) were thawed at room temperature. The contents of each well from the 7-day [^3^H]-hypoxanthine incorporation PRR assay (assay 1) were collected onto glass fiber filters using a Betaplate cell harvester (PerkinElmer). The dried filters were then placed in plastic foils containing 3.5 mL of scintillation fluid. Radioactivity signals (counts per minute) were measured using a Betaplate liquid scintillation counter (PerkinElmer). The contents of the two thawed sample aliquots were pipetted into the corresponding plates of assay 2 or assay 3 respectively, in four technical replicates. All wells of the 7- and 14-day *lacZ*/β-gal^SENSOR^ probe PRR assay were treated with β-gal^SENSOR^ probe (AquaSpark beta-D-galactoside, Biosynth AG, cat. #A-8169_P00) diluted in purified, sterile-filtered water containing MgCl_2_ to achieve final concentrations of 10 μM β-gal^SENSOR^ probe and 200 μM MgCl_2_ per well. The plates were then incubated at 37 °C for 30–60 min. Luminescence signals (counts/s) were measured using the multimode reader Spark from TECAN at 37 °C (exposure time 5 s).

### Parasite Reduction Ratio Assay Analysis and Evaluation

The criteria for determining whether a well was positive or negative for parasite growth differed for assay 1–3. For the 7-day [^3^H]-hypoxanthine incorporation PRR assay (assay 1) three criteria were used for parasite viability assessment: Discoloration of supernatant, discoloration of the filter mat and measured signal. A well was considered as positive for parasite growth if discoloration was visible in the assay supernatant or the filter mat (for overgrowing wells which will have no measurable signal), and/or if the measured signal exceeded the signal-to-noise cutoff value of three times the average of the measured negative control (uninfected RBCs). Different cutoff values were used to distinguish between positive and negative parasite growth in the 7-day (assay 2) and 14-day (assay 3) *lacZ*/β-gal^SENSOR^ probe PRR assay. These were calculated using the signal measured from the thawed samples representing the β-galactosidase concentration in the undiluted samples immediately after drug washout (at the 0 h incubation time point without drug pressure). The average of these signals was multiplied by the respective factors of 1.5 for assay 2 and 3 for assay 3. This value was then divided by the dilution factor used for the serial dilution to represent the calculated cutoff values for the initial titers, considering the stable β-galactosidase at the 0 h time point after drug washout, which resulted in higher signal levels within the initial dilution titers. The cutoff value for signal vs noise was calculated by multiplying the average of the measured negative control (uninfected RBCs) by three. For each titer level, the higher of the two cutoff values was determined. If the measured signal in a well was above the greater of these two cutoff values, the well was considered positive for parasite growth. Borderline positive values were considered negative if the two titer values above were negative and if the previous time point had already been deemed completely negative for parasite growth.

The number of viable parasites was extrapolated based on the number of wells with positive parasite growth (n) and the dilution factor (X) of the serial dilution, using the following formula: P_viable_ = X^*n*–1^ ^11,14^ (e.g.: parasite growth was measured at four titer levels → P_viable_ = 4^4–1^ = 64 parasites). As the experiment is started with a theoretical parasite number of 10^5^ parasites per well if untreated, final results were normalized so that the 0 h sample corresponded to 10^5^ parasites (for the 0 h plate following a 4-fold dilution, 8–10 titer levels are normally measured with parasite growth). If calculated parasite number based on experimental data for the 0 h plate corresponded to the theoretical 10^5^ parasites per well the normalization factor was close to 1. Values above 1 indicate that the experimental readout and back-extrapolation underestimated the starting inoculum of 10^5^ parasites. The raw data were analyzed using Microsoft Excel (Version 2505 build 16.0.18827.20102), and PD parameters were calculated using the R script developed by Walz et al.,^14^ (R version 4.5.1 (2025–06–13 ucrt) and RStudio version 2025.05.1 + 513). For the time-killing profile of artemisinin generated by assay 1, PD parameters were calculated in Excel (Version 2505 build 16.0.18827.20102): log_10_(PRR) = −48 h * slope of linear regression equation between 0 and 24 h; PCT_99.9%_ = (2- intercept of linear regression equation between 0 and 24 h)/slope of linear regression equation between 0 and 24 h. The lag time was examined visually.

### Drug Stability and Washout Controls

To assess drug stability within 24 h, the following samples were collected: a 0 h reference sample of the drug solution used to start the PRR assay and drug-containing supernatant before the drug removal by several washing steps 24 h after initialization of the PRR assay treatment. For the washout control, supernatant was collected after the final centrifugation step of the drug removal by the washing process and diluted in CM according to the procedure for the real sample, prior to serial dilution. Both controls (stability and washout) were tested using an IC_50_ assay as described in the section IC_50_ assay - [^3^H]-hypoxanthine incorporation, using the transgenic strain F12^*lacZ*^. The main difference from the standard IC_50_ assay was that the stability and washout samples were serially diluted in CM rather than in hypoxanthine-deficient medium.

The drug was considered stable if the fold change in IC_50_ between 24 and 0 h was less than 1.5. Drug washout was considered successful if the growth of parasites treated with washout control was comparable to that of the positive control in the IC_50_ assay (i.e., untreated parasites), meaning there was less than a 20% deviation between the two.^14^

## Abbreviations

PRR: parasite reduction ratio
IC_50_: half-maximal inhibitory concentration
PD: pharmacodynamic
PCT_99.9_%: parasite clearance time after which 99.9% of initial parasites have been cleared
*E*_max_: maximum effect
PRR v2: parasite reduction ratio version 2 assay published by Walz et al.
RBC(s): red blood cell(s)
CM: culture medium
HRP-2 ELISA: histidine-rich protein 2 sandwich enzyme-linked immunosorbent assay
h: hour(s)
SD, standard deviation DMSO: dimethylsulfoxide.
